# Correction: Turchi et al. CELF2 Sustains a Proliferating/OLIG2+ Glioblastoma Cell Phenotype via the Epigenetic Repression of SOX3. *Cancers* 2023, *15*, 5038

**DOI:** 10.3390/cancers17091583

**Published:** 2025-05-07

**Authors:** Laurent Turchi, Nathalie Sakakini, Gaelle Saviane, Béatrice Polo, Mirca Saras Saurty-Seerunghen, Mathieu Gabut, Corine Auge Gouillou, Vincent Guerlais, Claude Pasquier, Marie Luce Vignais, Fabien Almairac, Hervé Chneiweiss, Marie-Pierre Junier, Fanny Burel-Vandenbos, Thierry Virolle

**Affiliations:** 1 CNRS, INSERM, Institut de Biologie Valrose, Team INSERM “Cancer Stem Cell Plasticity and Functional Intra-tumor Heterogeneity”, Université Côte D’Azur, 06107 Nice, France; laurent.turchi@unice.fr (L.T.); nvs30@cam.ac.uk (N.S.); saviane.gaelle@hotmail.fr (G.S.); beatrice.polo@univ-cotedazur.fr (B.P.); almairac.f@chu-nice.fr (F.A.); burel-vandenbos.f@chu-nice.fr (F.B.-V.); 2 DRCI, CHU de Nice, 06107 Nice, France; 3 CNRS UMR8246, INSERM U1130, Neuroscience Paris Seine-IBPS Laboratory, Team Glial Plasticity and NeuroOncology, Sorbonne Université, 75252 Paris, France; sarasmsaurty@gmail.com (M.S.S.-S.); herve.chneiweiss@inserm.fr (H.C.); marie-pierre.junier@inserm.fr (M.-P.J.); 4 Stemness in Gliomas Laboratory, Cancer Initiation and Tumoral Cell Identity (CITI) Department, INSERM 1052, CNRS 5286, Centre Léon Bérard, 69008 Lyon, France; mathieu.gabut@inserm.fr; 5 Cancer Research Center of Lyon 1, Université Claude Bernard Lyon 1, 69100 Villeurbanne, France; 6 UMR 1253, iBrain, Inserm, Université de Tours, 37000 Tours, France; auge@univ-tours.fr; 7 CNRS, I3S, Université Côte d’Azur, 06560 Valbonne, France; guerlais@i3s.unice.fr (V.G.); claude.pasquier@unice.fr (C.P.); 8 CNRS, INSERM, Institut de Génomique Fonctionnelle, IGF, Université de Montpellier, 34090 Montpellier, France; marie-luce.vignais@igf.cnrs.fr; 9 Service de Neurochirurgie, Hôpital Pasteur, CHU de Nice, 06107 Nice, France; 10 Service d’Anatomopathologie, Hôpital Pasteur, CHU de Nice, 06107 Nice, France

In the original publication [1], errors were made in Figure 2. Panels 2A,C,E are incorrect and do not correspond to the original blot. The correct panels, in Figure 2, and caption are shown below. The authors apologize for any inconvenience caused and state that the scientific conclusions are not affected. This correction has been approved by the Academic Editor. The original publication has also been updated.

## Figures and Tables

**Figure 2:**
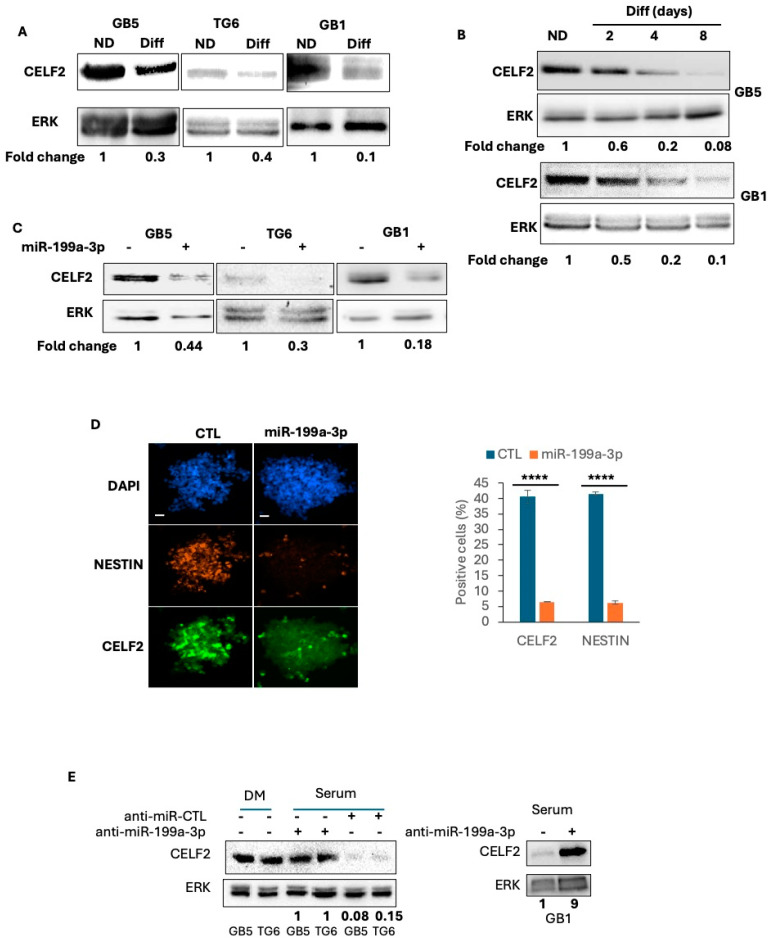
CELF2 expression and regulation in patient-derived GSCs. (**A**) Western blot analysis showing CELF2 expression in proliferating GSCs and their serum-differentiated counterparts (three days of differentiation). (**B**) Western blot showing CELF2 expression during the time course of GSC differentiation in serum medium. (**C**) GB5, TG6 and GB1 have been transfected by a synthetic miR-199a-3p or by a control non-relevant miRNA. CELF2 expression is revealed in both condition by western blot. (**D**) Immunofluorescence showing the expression of CELF2 or Nestin (marker of GSCs) in GB5 stably expressing a synthetic miR-199a-3p or a control non-relevant sequence. Scale = 100 µM. Histogram on the right displays the quantification of the number of positive cells (*p* value < 0.0001 ****). (**E**) GB5, TG6 and GB1 have been transfected by an anti-miR-199a-3p (synthetic sequence) or by a non-relevant sequence as anti-miRNA control. CELF2 expression is revealed by western blot.
