# Supplementary material for: CELF2 Sustains a Proliferating/OLIG2+ Glioblastoma Cell Phenotype via the Epigenetic Repression of SOX3
Source: Cancers (Basel). 2023 Oct 18;15(20):5038. doi: 10.3390/cancers15205038 (PMC10605641; doi:10.3390/cancers15205038)
Supplement: Supplementary file 1 [file cancers-15-05038-s001.zip › cancers-2483116-supplementary.pdf]

Supplemental figure legends

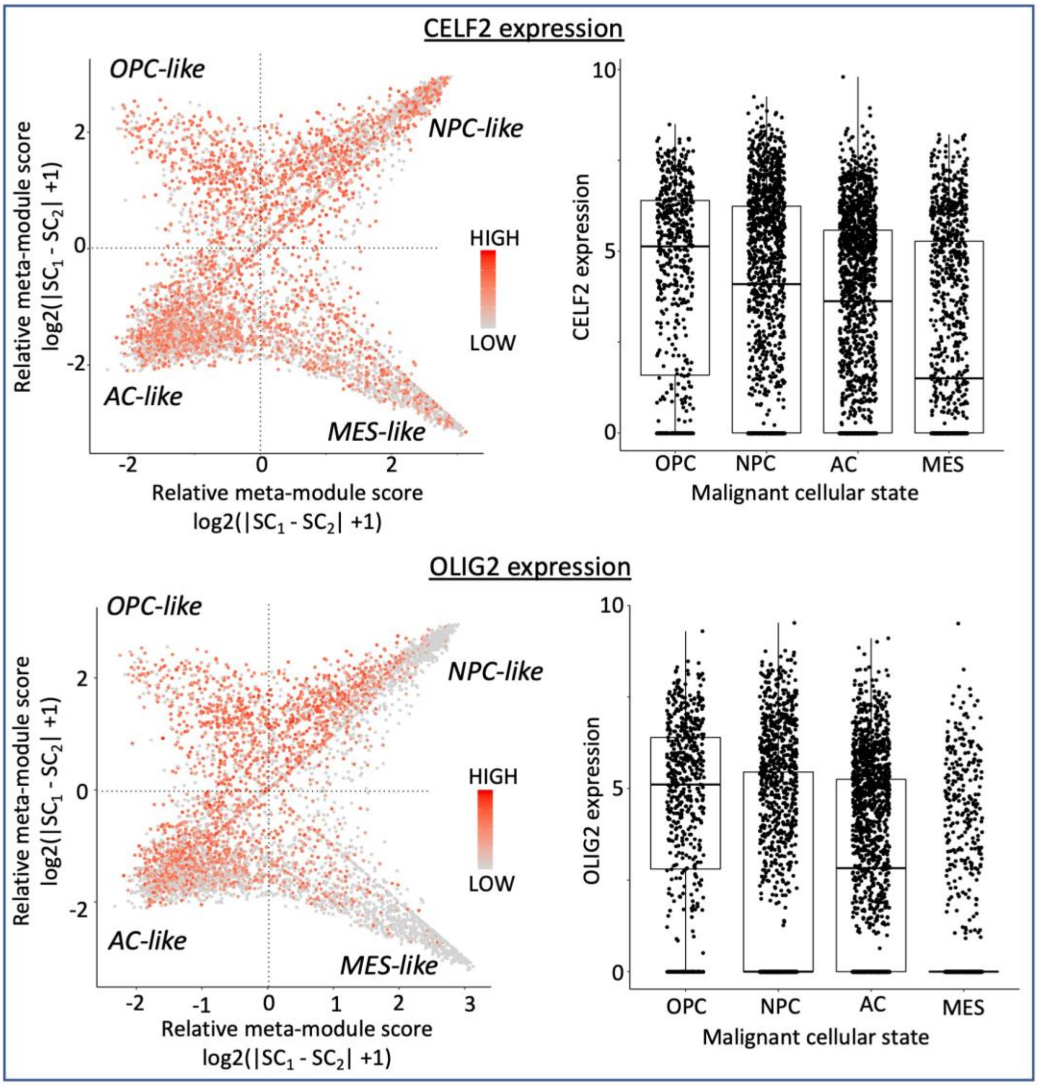

Figure S1. 2D representation of malignant cellular states as identified by Neftel and colleagues (Neftel et al, 2019) [9]. Cells colored by CELF2 or OLIG2 expression level. Boxplot showing CELF2 or OLIG2 expression in different malignant cellular states. Each group mean is different from one another (Kruskal-Wallis test and pairwise comparisons using Mann-Whitney test, BH-adjusted pval < 0.01).

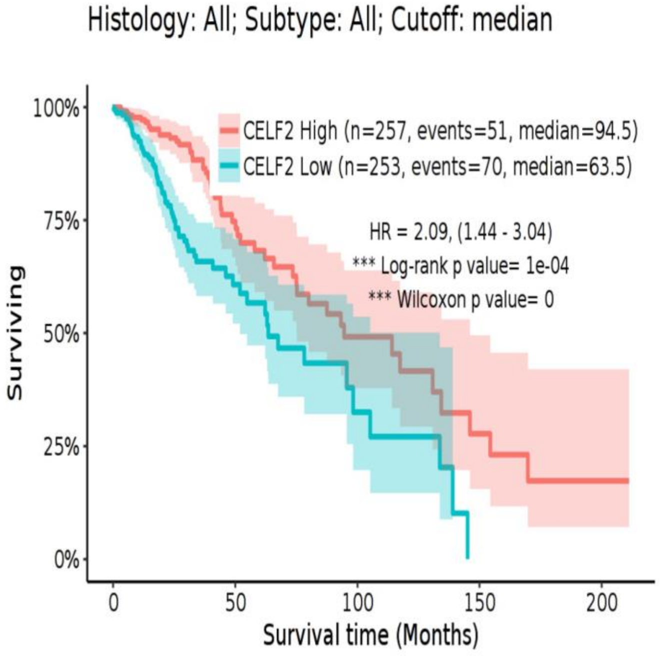

Figure S2. Survival analysis using the TCGA dataset, showing the CELF2 prognosis value in low grade glioma. The analysis was performed using the Gliovis interface (http://gliovis.bioinfo.cnio.es).

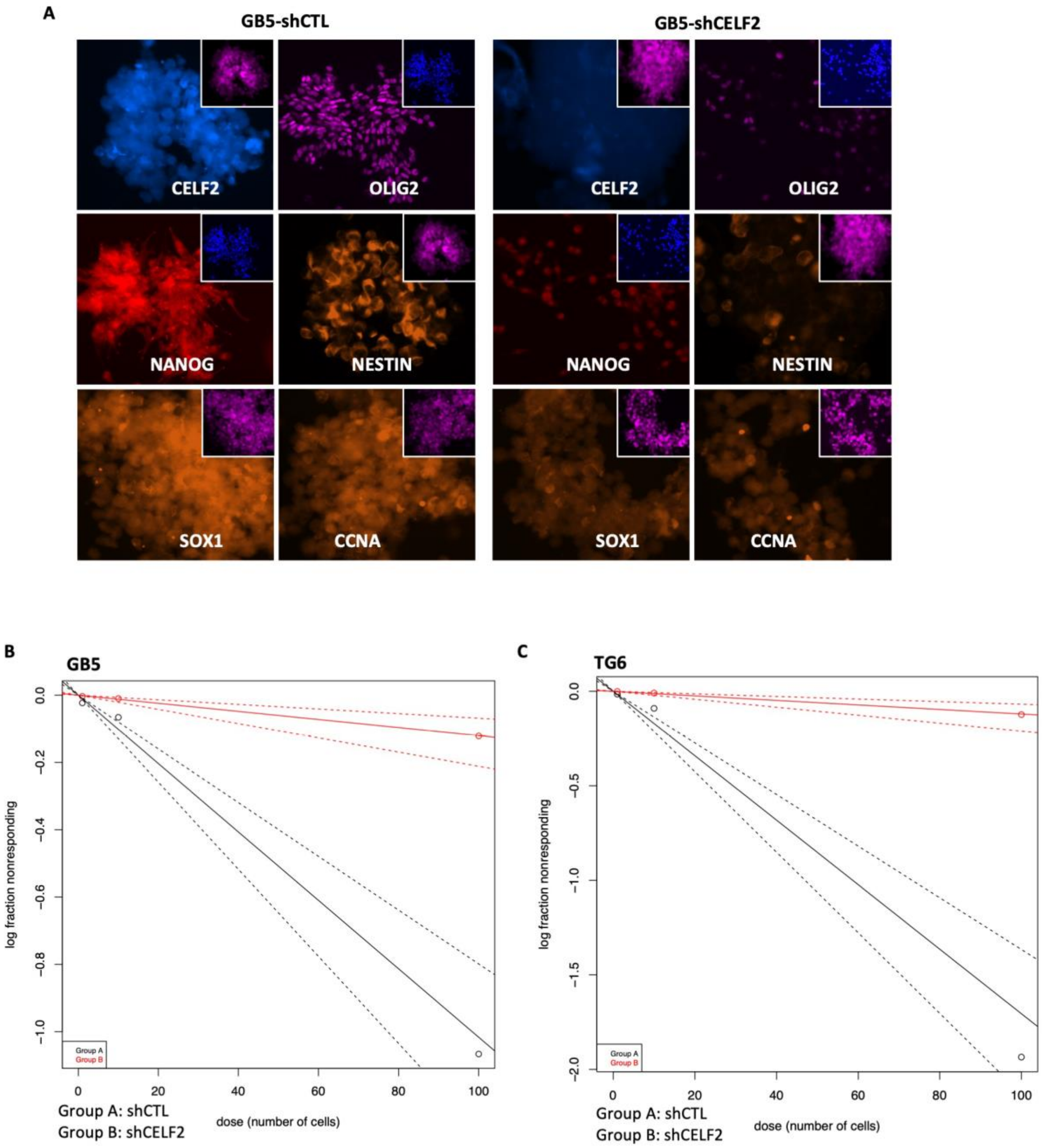

Figure S3. (A) Immunofluorescence showing CELF2, OLIG2, NANOG, NESTIN, SOX1, CYCLINA1 (CYCA) expression in GB5-shCTL and in GB5-shCELF2. (B-C) Limited dilution assay 100, 10 and 1 cell per well (ELDA analysis) performed on GB5 and TG6 cells invalidated (shCELF2) or not (shCTL) for CELF2 expression.

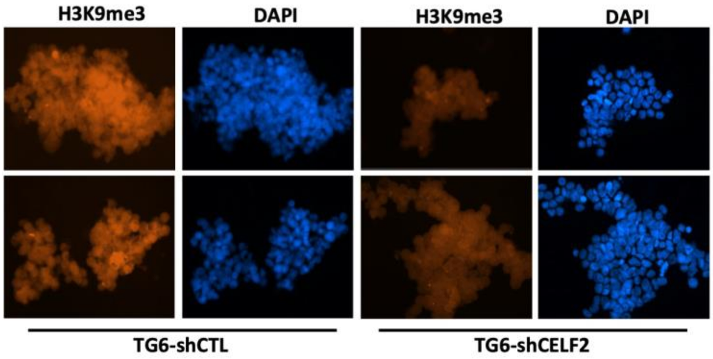

Figure S4. Immunofluorescence showing H3K9me3 expression in TG6-shCTL and in TG6-shCELF2.

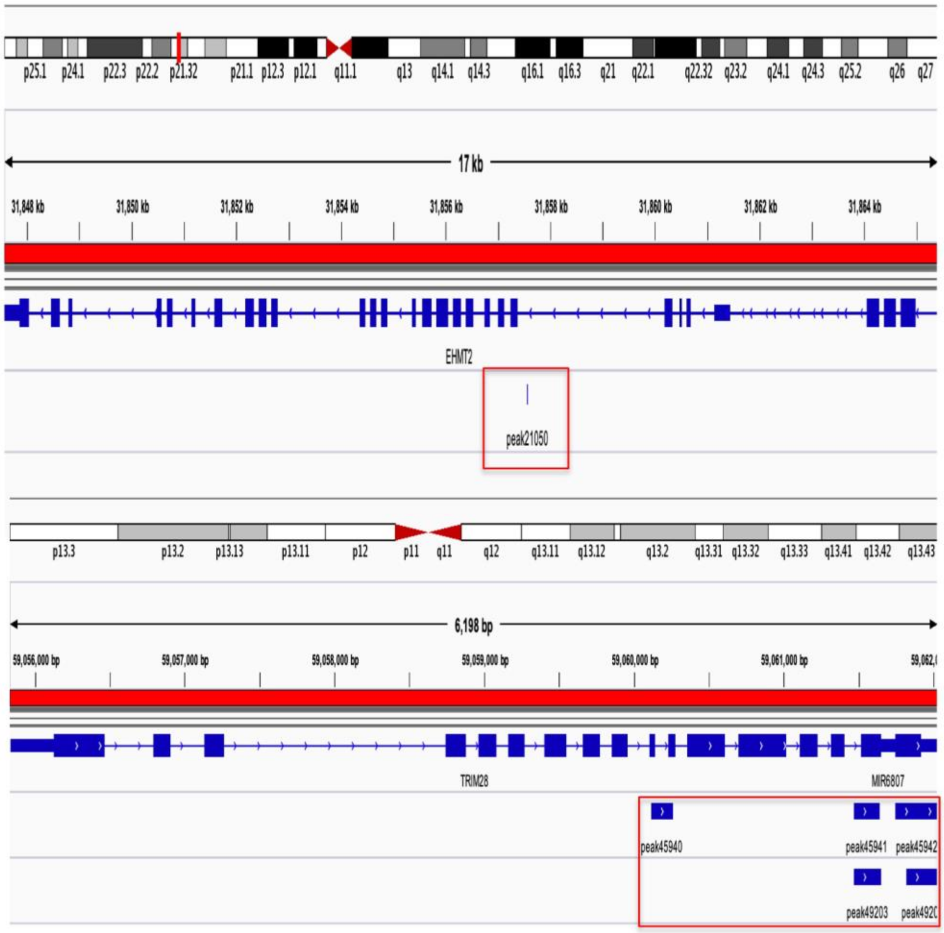

Figure S5. Genomic map showing the positions of CELF2 binding sites in TRIM28 and G9A mRNA as previously identified in CLIPseq analysis [40].

Figure S6. Whole western blot

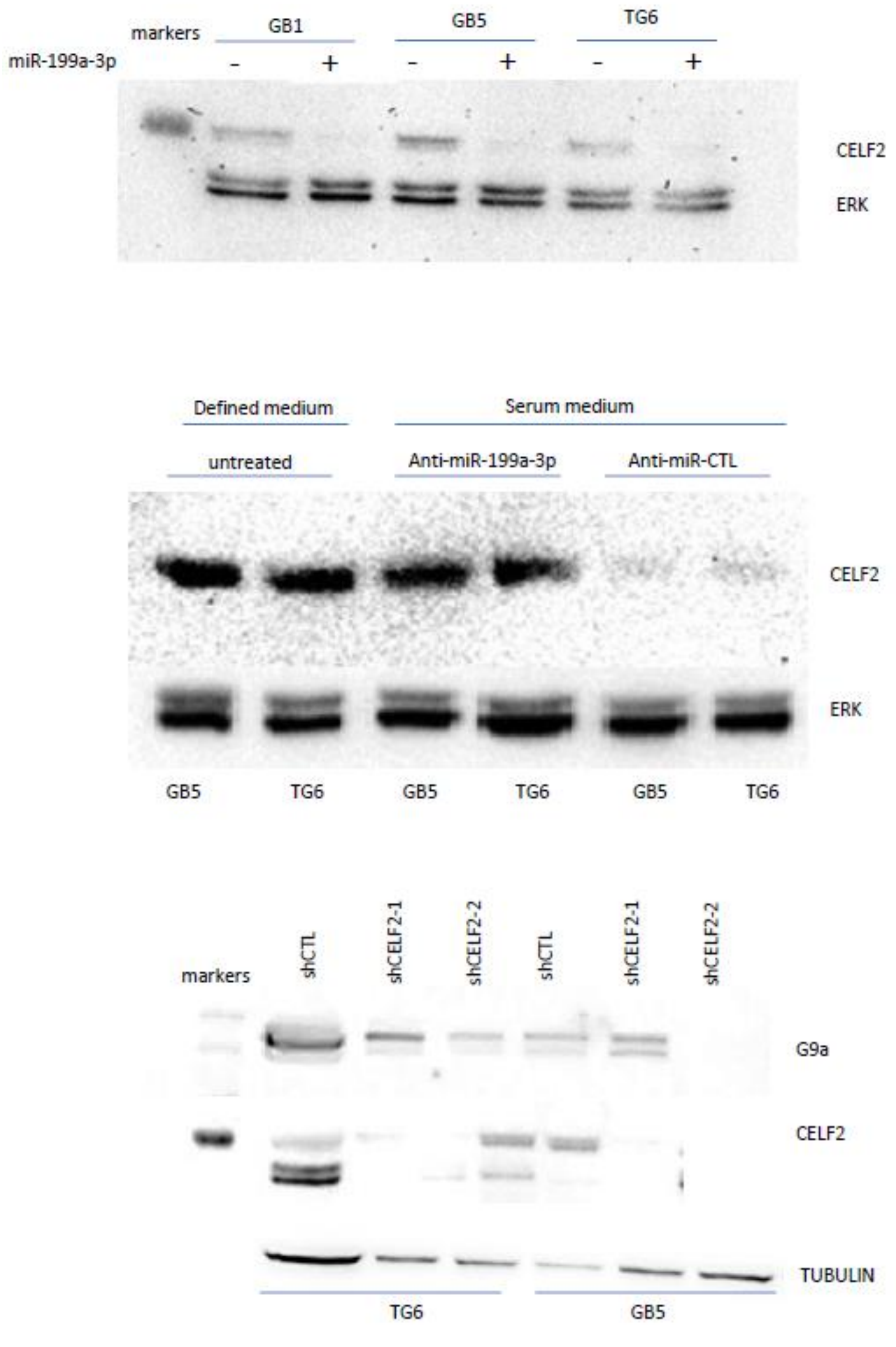

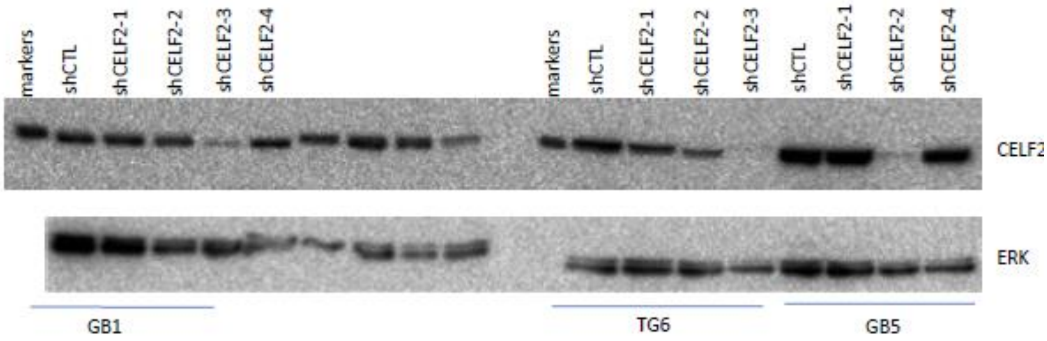
markers
shCTL
shCELF2-1
shCELF2-2
shCELF2-3
shCELF2-4
markers
shCTL
shCELF2-1
shCELF2-2
shCELF2-3
shCTL
shCELF2-1
shCELF2-2
shCELF2-4
CELF2
ERK
GB1
TG6
GB5

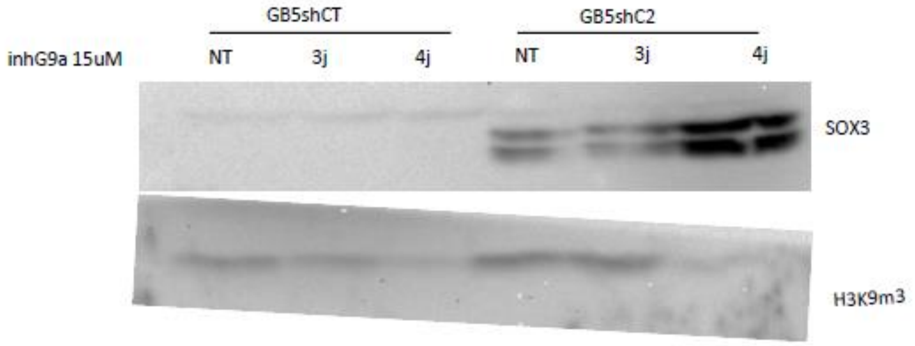
GB5shCT
GB5shC2
inhG9a 15uM
NT
3j
4j
NT
3j
4j
SOX3
H3K9m3

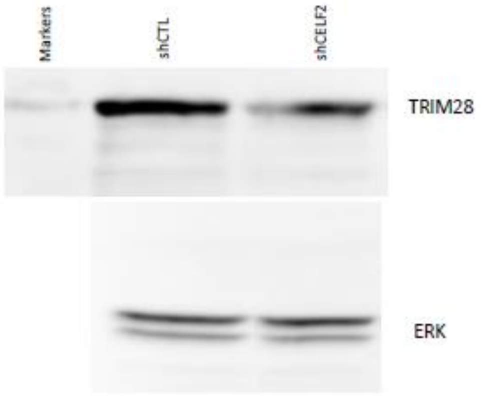
Markers
shCTL
shCELF2
TRIM28
ERK

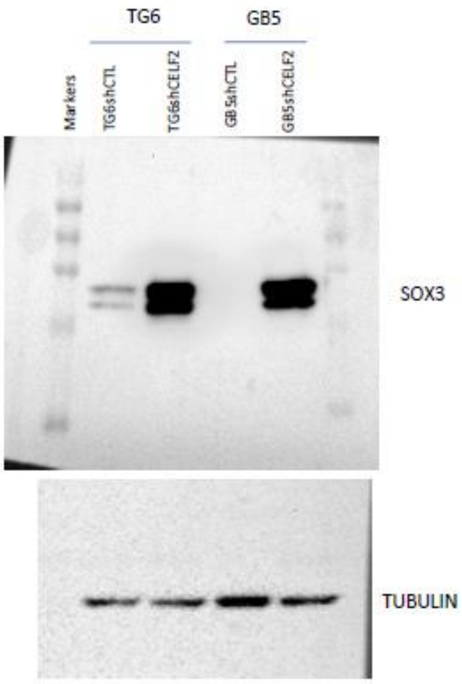
TG6
GB5
Markers
TG6shCTL
TG6shCELF2
GB5shCTL
GB5shCELF2
SOX3
TUBULIN

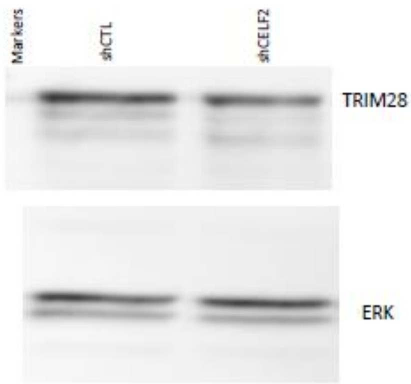
Markers
shCTL
shCELF2
TRIM28
ERK

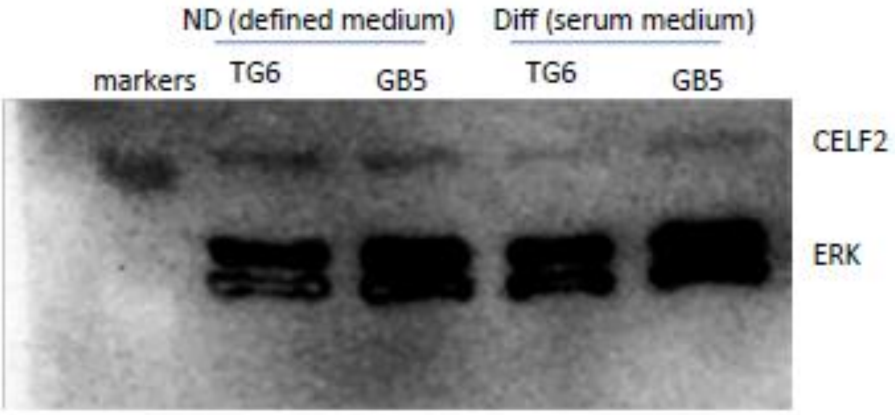

	mean expression				variance of expression				% detection			
	OPC-like	NPC-like	AC-like	MES-like	OPC-like	NPC-like	AC-like	MES-like	OPC-like	NPC-like	AC-like	MES-like
CELF2	4.26	3.57	3.21	2.58	7.18	8.72	7.14	7.39	80.34	66.94	68.49	56.49
OLIG2	4.42	2.53	2.79	1.07	6.5	8.65	7.05	4.26	82.18	46.27	58.65	24.03
SOX3	0.4	0.15	0.32	0.25	1.42	0.65	1.22	1.01	11.6	3.63	8.51	6.22

Supplemental Table S1: primer list used in the RIP experiments displayed in figure 4G.

Target mRNA	CELF2 position	Primers	Sequence
TRIM28	PEAK 45940	P40M1 S	GGTGAGTGGGTCTGCCTAGT
		P40M1 AS	GGCTCTGCACTTGAGTAGGG
		P40M2 S	CTATGGCTTTGGGTCAGGTG
		P40M2 AS	AAAGAAGACTGGCAGGTTGG
	PEAK 45941	P41M1 S	ACCCTGGATCTGACCCTGA
		P41M1 AS	ATCCTGGGCAAACTCCTGT
		P41M2 S	ACCCTGGATCTGACCCTGA
		P41M2 AS	ACATCCTGGGCAAACTCCT
	PEAK 49204	P04 S	TGAGTTCCCAGGAGCTGTCT
		P04 AS	GATGGGGTGACAGGACAGAG
	PEAK 45942	P42 S	CAGACGTGCAGTCCATCATC
		P42 AS	CAGCACAGCAGAGAACTTGG
EHMT2	PEAK 21050	G9aM1 S	ACCAAGATCTGTGCACATTGC
		G9aM1 AS	TGTGGGTAGCAGAGGAGACA

Supplemental Table S2: list of the 104 genes identified in figure 5D.
